# Susceptibility artefact correction using dynamic graph cuts: Application to neurosurgery

**DOI:** 10.1016/j.media.2014.06.008

**Published:** 2014-07-05

**Authors:** Pankaj Daga, Tejas Pendse, Marc Modat, Mark White, Laura Mancini, Gavin P. Winston, Andrew W. McEvoy, John Thornton, Tarek Yousry, Ivana Drobnjak, John S. Duncan, Sebastien Ourselin

**Affiliations:** a Centre for Medical Image Computing, University College London, London, UK; b Department of Clinical and Experimental Epilepsy, Institute of Neurology, University College London, London, UK; c National Hospital for Neurology and Neurosurgery, UCLH NHS Foundation Trust, London, UK; d Dementia Research Centre, Institute of Neurology, University College London, London, UK

**Keywords:** Phase unwrapping, Susceptibility, Magnetic resonance imaging, Graph cuts, Image-guided neurosurgery

## Abstract

Echo Planar Imaging (EPI) is routinely used in diffusion and functional MR imaging due to its rapid acquisition time. However, the long readout period makes it prone to *susceptibility artefacts* which results in geometric and intensity distortions of the acquired image. The use of these distorted images for neuronavigation hampers the effectiveness of image-guided surgery systems as critical white matter tracts and functionally eloquent brain areas cannot be accurately localised. In this paper, we present a novel method for correction of distortions arising from susceptibility artefacts in EPI images. The proposed method combines fieldmap and image registration based correction techniques in a unified framework. A phase unwrapping algorithm is presented that can efficiently compute the B_0_ magnetic field inhomogeneity map as well as the uncertainty associated with the estimated solution through the use of dynamic graph cuts. This information is fed to a subsequent image registration step to further refine the results in areas with high uncertainty. This work has been integrated into the surgical workflow at the National Hospital for Neurology and Neurosurgery and its effectiveness in correcting for geometric distortions due to susceptibility artefacts is demonstrated on EPI images acquired with an interventional MRI scanner during neurosurgery.

## 1 Introduction

Echo Planar Imaging (EPI) provides high temporal resolution and is routinely used in functional magnetic resonance imaging (fMRI) and diffusion weighted imaging (DWI) sequences. In recent years, interventional MRI (iMRI) is fast emerging as a popular imaging choice for image-guided neurosurgery. The high spatial resolution, excellent soft tissue contrast and the lack of ionising radiation makes iMRI an attractive imaging option for guiding interventions. Furthermore, along with conventional structural imaging, current commercial iMRI scanners can also perform diffusion-weighted and functional imaging which allows for intra-operative visualisation of eloquent brain areas and critical white matter tracts along with the surgical target areas. Modern iMRI scanners use EPI sequences to acquire DWI images during neurosurgery, which can be then used for localisation of critical white matter tracts that lie close to the surgical field. EPI performs fast imaging by sampling the selected slice with one excitation pulse and fast gradient blipping. However, this results in very low bandwidth in the phase encoding direction, which makes EPI images highly susceptible to small perturbations of the magnetic field, giving rise to various artefacts because of the magnetic field inhomogeneities. The primary source of *susceptibility artefacts* is the difference in magnetic susceptibility between various tissues being imaged. In the context of neuroimaging, this leads to severe geometric and intensity distortions in areas like the brain stem, frontal and temporal lobes. The distortions are especially severe as the surgically resected cavity contains air and induces high susceptibility differences by introducing an air-tissue interface and leading to large distortions around the area of resection.

The National Hospital for Neurology and Neurosurgery (NHNN) in London is one of the leading centres for surgical management of focal epilepsy in the UK. Epilepsy is a common and debilitating neurological disorder and around one-third of patients with focal epilepsy are refractory to treatment with anti-epileptic drugs. The majority have temporal lobe epilepsy for which anterior temporal lobe resection is a well-established and effective treatment (Wiebe et al., 2001). An important source of morbidity during anterior temporal lobe resection arises due to damage to a white matter tract called the optic radiation during the surgical intervention. This can lead to severe visual field deficits that can result in a significant loss of vision, even if the patient is seizure free after surgery. Since the optic radiation cannot be identified visually during surgery without appropriate imaging, its accurate localisation and real-time display could be crucial in improving the surgical outcome for patients undergoing anterior temporal lobe resection. Recent studies have shown that the use of diffusion weighted EPI images along with structural images in non-rigid registration algorithms can accurately localise brain structures of interest during neurosurgical procedures (Daga et al., 2012; Winston et al., 2011). There is also an interest in performing tractography on interventional DWI images to segment white matter structures of interest (Chen et al., 2009; Sun et al., 2011; Andrea et al., 2012; Cardoso et al., 2012). Hence, it is important to accurately compensate for susceptibility artefacts to be able to efficiently use EPI images for neuronavigation.

Correction of susceptibility induced distortions in EPI images falls under two broad categories: fieldmap estimation and non-linear image registration. The fieldmap estimation approach is the estimation of B_0_ magnetic field inhomogeneity at every voxel from phase images acquired at different echo times as demonstrated in (Jezzard and Balaban, 1995; Funai et al., 2008; Jenkinson, 2003). It was shown in Wu et al. (2008) that correction of susceptibility artefacts by fieldmaps can be inaccurate in regions of high field inhomogeneity. This is especially critical when correcting EPI images that are acquired using interventional MRI during a neurosurgical procedure. The area of resection often lies in close proximity to critical white matter tracts and as the neurosurgical procedure progresses, information on the exact current location of the tract is beneficial for surgical planning. However it is exactly at the resection margin with the brain/air interface that the B_0_ magnetic field is most inhomogeneous and produces maximum geometric and intensity distortions. A popular alternative to fieldmaps is to use intensity based non-rigid image registration techniques to register the distorted EPI image to a high resolution undistorted T1-weighted MRI (Kybic et al., 2000; Merhof et al., 2007; Tao et al., 2009; Huang et al., 2008). However, the EPI images acquired interventionally have low signal-to-noise ratio and suffer from various artefacts which makes intensity based image registration challenging. A recent work by Irfanoglu et al. (2011), an extension of Jenkinson et al. (2004), proposed the generation of fieldmap estimates from structural images, which was then used to sample a non-uniform B-spline grid for an elastic registration based correction step. This procedure, however, is difficult to apply in the interventional setting due to the complex physical environment around the resection area and the need for tissue segmentation maps. Registration based approaches which require acquisition of an additional EPI image have also been proposed (Studholme et al., 2000; Ruthotto et al., 2012).

Additionally, any proposed solution must work within the stringent time constraints of a neurosurgical procedure. The current patient transfer time from the intra-operative scanner, after an imaging session, to the surgical bed at NHNN is between 7 and 9 min. *All* image analysis tasks must be performed within this time window to ensure no extra time is added to the surgery.

**Fig. 1:**
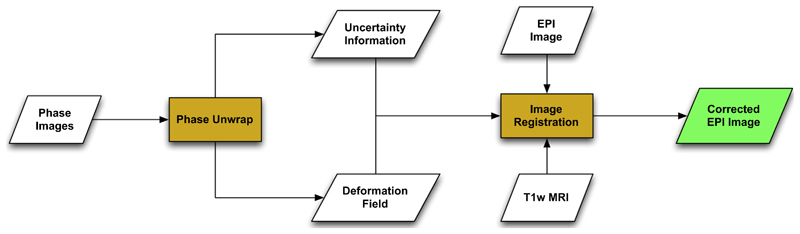
The proposed workflow for correction of susceptibility artefacts in EPI images acquired during neurosurgery. The field map is calculated using the acquired phase images which are unwrapped using the proposed algorithm. The estimated deformation field and the uncertainty information associated with the phase unwrapping step is used to initialise the image registration step where the EPI image and the corresponding undistorted T1-MRI image is used as the source and the target images respectively. The registration step is selectively driven in regions of high uncertainty to improve the results in areas where the field map might have resulted in a sub-optimal solution.

This work meets the aforementioned challenges by combining the fieldmap and image registration based correction approach in a unified scheme. The main idea behind the proposed work is a novel phase unwrapping algorithm that can also compute the uncertainty associated with the estimated fieldmap. The deformation field generated from the fieldmap correction step and the associated uncertainty measure are used to initialise and guide a subsequent image registration step. The overall workflow can be visualised as Fig. 1. The proposed work is also suitable to be used within the neurosurgical environment due to use of fast optimisation provided by graph cuts.

The main contributions of this work are:

- A phase unwrapping algorithm using dynamic graph cuts that also determines the uncertainty associated with the estimated solution.
- A registration algorithm that can be utilise the uncertainty information estimated from the phase unwrapping step to refine the results in areas where the fieldmap estimates are likely to be incorrect.
- Demonstrate the use of the proposed method during neurosurgery at NHNN, London on 13 patients within the time constraints of the intervention.

The paper is organised as follows: we describe our iMRI setup and NHNN in Section 2. Section 3 describes the noise model in the MRI phase images and highlights the assumptions of our phase model. Section 4 describes the graph cuts based phase unwrapping method. Section 5 describes how uncertainty information can be computed from the phase unwrapping step and can be used with an image registration method to further improve results. Validation on synthetic and clinical datasets are described in Sections 6.1 and 6.2 respectively.

## 2 Interventional MRI setup

**Fig. 2:**
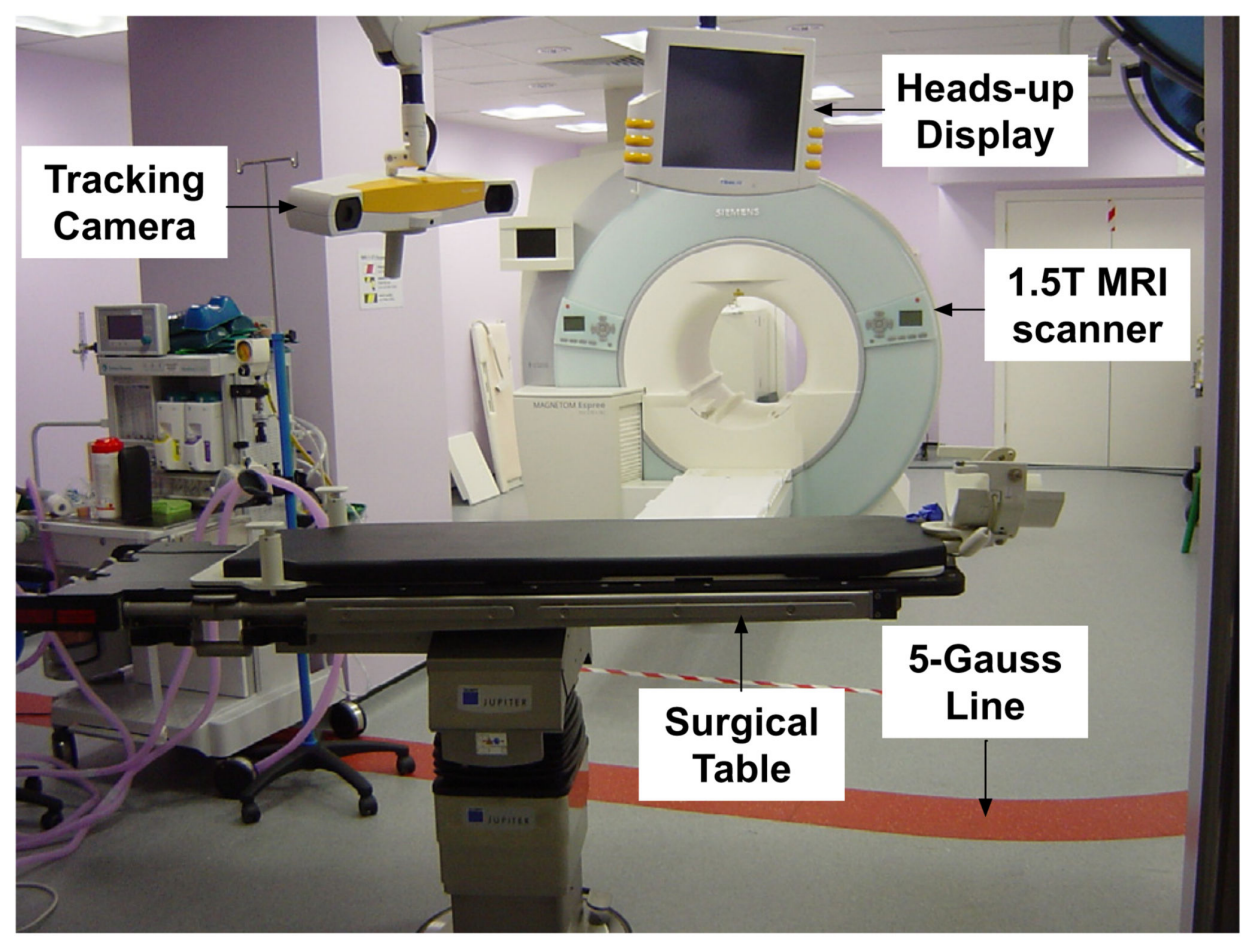
Interventional MRI surgical suite at the National Hospital for Neurology and Neurosurgery with a 1.5 T MR scanner and neuronavigation equipment. The surgical table interfaces with the scanner to enable the patient to be moved in and out of the scanner efficiently during surgery.

The iMRI setup at NHNN, London consists of a 1.5 T Siemens (Erlangen, Germany) Espree MRI machine. There is a dedicated operating room 8 channel MR head coil which incorporates a surgical headrest. The operating table is fitted with an MR compatible head-holder and is placed outside the 5 Gauss line during surgery which enables the surgeons to perform the procedure using standard non-MR-compatible surgical instruments. The table can interface with the MR scanner to allow the patient to be moved in and out of the scanner for intra-operative imaging. The facility is equipped with a BrainLAB VectorVision^®^ Sky neuronavigation system which provides real-time tracking of surgical markers and tools, *global* image registration and visualisation facilities. The operating room is also equipped with an Opmi Pentero confocal surgical microscope (Carl Zeiss), supporting the injection of colour overlays from the navigation system. The location of the microscope’s focal point is tracked using the navigation system and an array of four infra-red reflectors mounted on the microscope’s optical head. A snapshot of the iMR surgical room is shown in Fig. 2.

The current interventional workflow at NHNN acquires iMR images at two timepoints: after performing the craniotomy and after the temporal pole resection. Structural and diffusion weighted images are acquired and are corrected for gradient non-linearities and susceptibility artefacts (using the proposed method). The images are then used as target images in a non-rigid image registration scheme presented in Daga et al. (2012), which uses both the structural and diffusion weighted images in a bivariate similarity measure. The deformation field obtained from the image registration step is used to propagate the pre-operatively parcellated white matter tracts to the intra-operative geometry for neuronavigation. Susceptibility artefacts create severe distortions around the area of resection in the diffusion weighted images and it is important to correct for them for accurate image registration. In addition, the neurosurgery environment is complex and there are stringent time constraints. As already mentioned, the patient transfer time after an imaging session to the surgical bed is between 7 and 9 min. The susceptibility correction and the image registration steps should be performed within this time window so as to not add extra time to surgery.

## 3 Noise in MRI phase images

iMRI scanners come in various configurations with a constant tradeoff between the signal to noise ratio and access to the patient. Hence, it is important to characterise the noise distribution in the iMRI setting as this can have a significant impact on the performance of the image analysis algorithms. The noise characteristics of MRI images were studied in detail by Gudbjartsson and Patz (1995). MRI phase images are reconstructed from the real and the imaginary images by calculating pixel by pixel the arctangent of their ratio. This is a nonlinear function and therefore the underlying noise distribution is not Gaussian anymore. The distribution of the phase noise, Δ*θ*, is given by Eq. (1).

(1) $$p(\Delta \theta )=\frac{1}{2\pi }e^{-A^{2}/2\sigma^{2}}\left[1+\frac{A}{\sigma }\sqrt{2\pi }\;\text{cos}\;\Delta \theta \;\text{exp}(A^{2}{\;\text{cos}\;}^{2}\Delta \theta /2\sigma^{2})\frac{1}{2\pi }\int_{-\infty }^{\frac{A\text{cos}\Delta \theta }{\sigma }}\text{exp}(-x^{2}/2)\text{d}x\right]$$

where *A* is the noise-free phase value and *σ* is the standard deviation of noise in the real and imaginary channels (the noise is assumed to be identically distributed in the two channels). The underlying general distribution of the phase noise is, therefore, non-Gaussian. However, if we consider the case when *A* = 0 i.e. in background image regions where there is only noise, the distribution simplifies to *p*(Δ*θ*) = 1/2*π* which corresponds to a uniform probability in all phase directions. Considering another case, where *A* ≫ *σ* i.e. image regions where the signal is significantly greater than noise, we also obtain a simpler distribution as:

(2) $$p(\Delta \theta )\approx \frac{1}{2\pi {\left(\sigma /A\right)}^{2}}\;\text{exp}\;\left(\frac{-\Delta \theta^{2}}{2{\left(\sigma /A\right)}^{2}}\right)$$

Hence, the phase noise distribution can be assumed to be additive zero mean Gaussian distributed when *A* ≫ *σ*. The signal to noise ratio in iMRI images is typically lower than conventional MRI images. However, the Gaussian assumption of noise distribution is appropriate even for fairly small signal to noise ratios as was shown by Gudbjartsson and Patz (1995). The phase unwrapping method presented in the next section formulated under this Gaussian noise distribution assumption.

## 4 Phase unwrapping

A popular method for estimating the magnetic field map is to use the phase difference between two MR images acquired at different echo times. The phase measurements at the two echo times can be used to generate the field map through Eq. (3) where Δ*B*_0_ (*i*) is the field inhomogeneity at a given voxel location *i*, *Θ*(*i*) is the phase difference measured over time Δ*TE* and *γ* is the gyromagnetic ratio. The phase evolution can be extracted from the difference of two echoes, which eliminates effects that are common to both images.

(3) $$\Delta B_{0}(i)={\left(2\pi \gamma \Delta TE\right)}^{-1}\Delta \Theta (i)$$

The one-dimensional displacement along the phase encode direction can be computed by multiplying the field map by the acquisition time as:

(4) $$\delta_{PE}(x,y,z)=\gamma \Delta B_{0}(x,y,z)T_{acq}$$

where *δ_PE_* (*x*, *y*, *z*) is the one-dimensional voxel displacements in the phase encode direction and *T_acq_* is the readout time for a slice of MR data.

Hence, accurate correction of susceptibility artefacts is contingent upon being able to accurately measure the phase at the different echo times. However, the phase images are uniquely defined only in the range of (−*π*, *π*] and the phase images need to be *unwrapped* at each voxel by an unknown integer multiple of 2*π* to obtain the true phase as in Eq. (5).

(5) $$\phi_{t}(i)=\phi_{w}(i)+2\pi k_{i}$$

where *ϕ_t_*(*i*) is the true phase at a given voxel *i*, *ϕ_w_*(*i*) is the wrapped phase and *k_i_* is the unknown integer multiple of 2*π* that needs to be estimated. In the absence of noise provided that the underlying field is spatially continuous, the only discontinuities that can occur in the measured phase image is due to wrapping itself. In that specific case, phase unwrapping is relatively easy to address. To unwrap, the phase difference between adjacent samples is calculated and if it is greater than *π*, phase wrapping has occured. In the absence of noise, the measured phase image can be correctly unwrapped provided that there are no discontinuities between adjacent voxels in the true phase image that are greater than *π*. While this algorithm is simple to implement, it can fail in areas with low signal to noise and these errors can propagate through the overall unwrapping process creating unwrapping failure over a large area.

To cope with this issue, we propose a Bayesian approach to the phase unwrapping problem. As we have described, phase unwrapping is an ill-posed problem in the presence of noise and becomes intractable without regularisation. Similar to Ying et al. (2006), the phase is modelled as a Markov Random Field (MRF) where the true phase *ϕ_t_* and the wrapped phase *ϕ_w_* are treated as random variables. The aim is to find the discrete label configuration *k* that gives the maximum a posteriori (MAP) estimate of the phase wraps as shown in Eq. (6). MRF is an intuitive choice for this problem as an individual voxel does not provide any information to perform the phase unwrapping and there is a need to specify spatial constraint and relationships among neighbouring voxels, which can be done conveniently through an MRF. Furthermore, there are computationally attractive options at our disposal to perform inference on such a system.

(6) $$\phi_{t}=\underset{k}{\text{max}\;}\underset{\text{Likelihood}}{\underset{︸}{P(\phi_{w}|\phi_{t})}}\;\underset{\text{Prior}}{\underset{︸}{P(\phi_{t})}}$$

The likelihood term in Eq. (6) is modelled as *δ*(*ϕ_w_* − *W*(*ϕ_t_*)), where *δ* is the delta function and *W*(*ϕ_t_*) is the wrapped true phase. This is ill-posed and additional constraints on the true phase are incorporated in terms of prior probabilities. The MR phase can be modelled as a piecewise smooth function where the smooth component is due to the inhomogeneities in the static MR field and the non-smooth component arises due to changes in the magnetic susceptibility at boundaries between tissues of different types. The spatial smoothness is enforced by modelling the true phase as a MRF and incorporating the smoothness model through a suitable potential function. In this work, we model the true phase as a six-neighbourhood pairwise MRF where the pairwise potential function used is the sum of square of difference of the true phase between adjacent neighbours. Owing to the MRF-Gibbs equivalance (Li, 1994), the phase unwrapping problem is to find the MRF labelling or configuration that minimises the energy *E*(*k*|*ϕ_w_*):

(7) $$E(k|\phi_{w})=\underset{k}{\text{argmin}}\sum_{i\in I}\sum_{\Omega }V(\Delta \phi_{t}^{i})$$

where *I* are the image voxels, *Ω* is the set of neighbours for a given voxel at location *i*. $V(\Delta \phi_{t}^{i})$ is the potential function defined on the difference potential between a voxel *i* and its neighbours in *Ω*. The unknown integer wraps are denoted by *k*. The following subsection describes how this integer constrained global optimisation problem can be efficiently solved using graph cuts.

### 4.1 Energy minimisation via graph cuts

Graph Cuts have emerged as a popular method for optimisation of such multi-label problems (Boykov et al., 2001; Kolmogorov and Zabin, 2004). A graph *G* = (*V*, *E*) consists of a set of vertices *V* and edges *E* and two special terminal vertices termed source *s* and sink *t*. A cut on such a graph is a partition of the vertices *V* into disjoint sets *S* and *T* such that *s* ∈ *S* and *t* ∈ *T*. The cost of the cut is then the sum of all edges going from *S* to *T* across the cut boundary. The main idea behind using graph cuts for finding the minimum energy configuration of an MRF is to construct a graph where there is one-to-one correspondence between the cuts on the graph and configurations of the MRF. Additionally, the total cost of a given cut should represent the energy of the corresponding MRF configuration. Hence, the task of finding the minimum energy configuration of the MRF in Eq. (7) is equivalent to finding the cut on the representative graph with the minimum cost. The minimum cost cut can be efficiently found by using the maximum flow algorithm (Ford and Fulkerson, 1962). An important advantage of Graph Cuts is that the maximum flow is a low-order polynomial time algorithm, which makes it computationally very efficient and suitable for use in neurosurgical applications.

It was shown in Kolmogorov and Zabin (2004) that an energy function with the form of Eq. (8), where *E^i^* is the unary energy term and *E^ij^* is the pair-wise energy term, can be represented by a graph as long as each pair-wise term *E^ij^* satisfies the inequality in Eq. (9). The proposed energy function of Eq. (7) has the structure of Eq. (8) with null unary data term.

(8) $$E(x_{1},x_{2},\ldots ,x_{u})=\sum_{i=1}^{u}E^{i}(x_{i})+\sum_{i=1;i<j}^{u}E^{ij}(x_{i},x_{j})$$

(9) $$E^{ij}(0,0)+E^{ij}(1,1)⩽E^{ij}(0,1)+E^{ij}(1,0)$$

Phase unwrapping using graph cuts was first proposed by Bioucas-Dias and Valadao (2007) for interferometric applications. They showed that for convex pairwise potential functions, an iterative minimisation algorithm can be constructed using graph cuts. If the pairwise energy term *V* is convex and if the minima of *E*(*k*|*ϕ_w_*) is not reached, a binary image *δ* ∈ (0, 1) exists such that *E*(*k* + *δ*|*ϕ_w_*) < *E*(*k*|*ϕ_w_*). For brevity let us consider the problem in one dimension and assume a two neighbourhood MRF system i.e. we only consider a single pair of neighbours. This can be easily extended to multiple dimensions by simply adding the terms corresponding to the neighbours in the other spatial dimensions. Let $k_{t+1}^{i}=k_{t}^{i}+\delta^{i}$ be the wrap count at time *t* + 1 at voxel *i*. Then, we have Eq. (10) where Δ*ϕ_t_* is the difference in the true phase between the MRF neighbours.

(10) $$\Delta \phi_{t}=2\pi (k_{t+1}^{i}-k_{t+1}^{i-1})+(\phi_{w}^{i}-\phi_{w}^{i-1})$$

After algebraic manipulation of Eq. (10), we can rewrite the energy function as Eq. (11).

(11) $$E(k_{t}+\delta |\phi_{w})=\underset{n}{\text{argmin}}\sum_{i\in I}\sum_{s\in \Omega }V(2\pi (\delta^{i}-\delta^{i-1})+2\pi (k_{t}^{i}-k_{t}^{i-1})+(\phi_{w}^{i}-\phi_{w}^{i-1}))$$

Now considering the terms in Eq. (9), we have:

$$\begin{array}{l} E\left(0,0\right)=V\left(t\right)\\ E\left(1,1\right)=V\left(t\right)\\ E\left(1,0\right)=V\left(2\pi +t\right)\\ E\left(0,1\right)=V\left(-2\pi +t\right) \end{array}$$

where

$$t=2\pi (k_{t}^{i}-k_{t}^{i-1})+(\phi_{w}^{i}-\phi_{w}^{i-1})$$

As *V* is convex, *E^ij^*(0, 0) + *E^ij^*(1, 1) ⩽ *E^ij^*(0, 1) + *E^ij^*(1, 0) or *V*(2*π* + *t*) + *V*(−2*π* + *t*) ⩾ 2 × *V*(*t*). Hence, the proposed energy term can be represented by a graph.

**Fig. 3:**
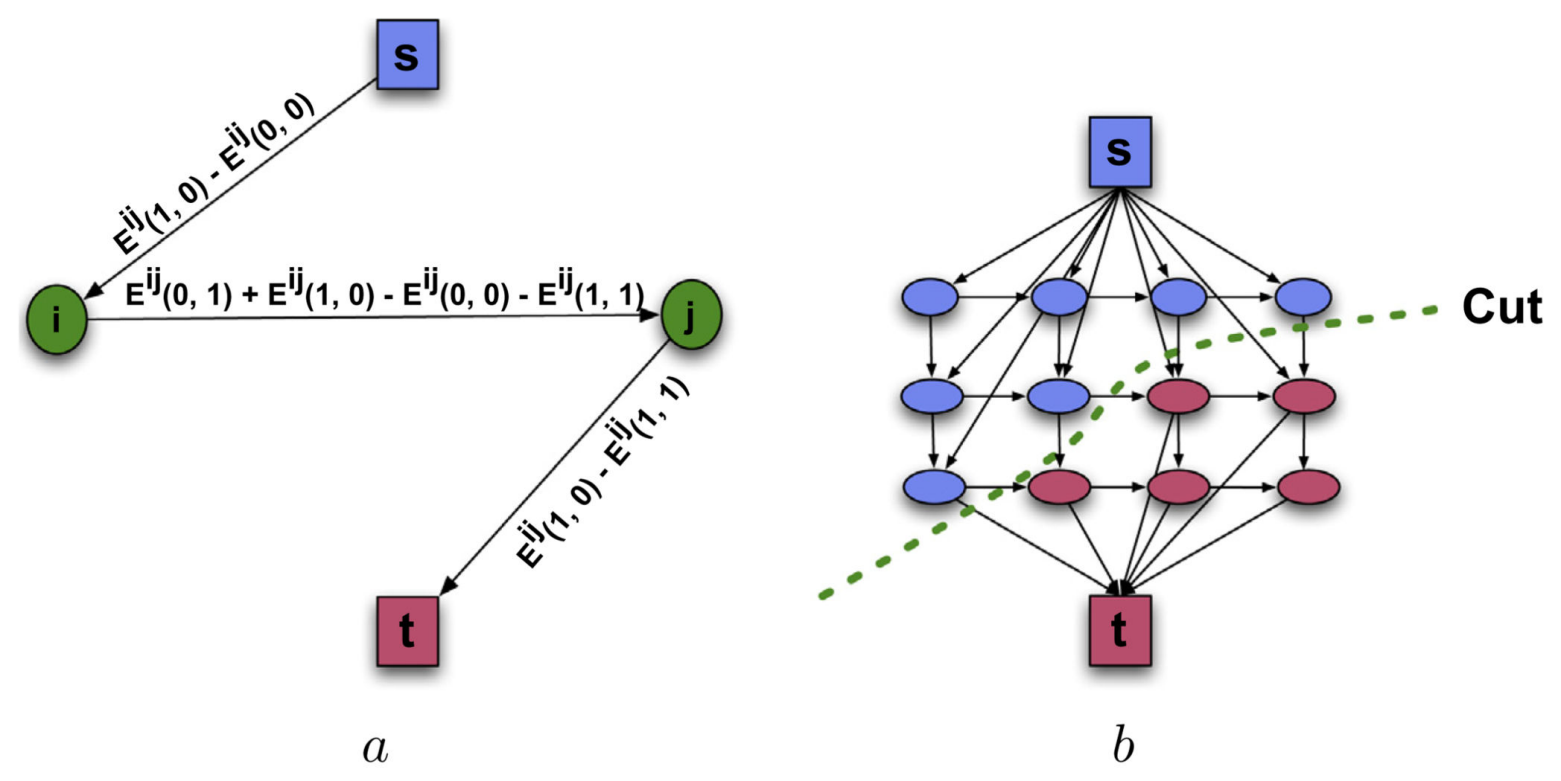
Graph Construction. (a) Shows the construction of the elementary graph for a single pairwise term between two neighbouring voxels i and j when *E^ij^*(1, 0) − *E^ij^*(0, 0) > 0 and *E^ij^*(1, 0) − *E^ij^*(1, 1) > 0. While there are no constraints on the edges connected to the terminal nodes (highlighted by s and t), the edges between data nodes must be non-negative and satisfy the submodularity constraint of Eq. (9). (b) Shows the building of the graph by merging the elementary graphs together. After the graph is constructed, maximum flow algorithm can be used to find the minimum cut (denoted by the dashed line) on the graph.

Fig. 3(a) shows how an elementary graph between two MRF neighbours is constructed when *E^ij^*(1, 0) − *E^ij^*(0, 0) > 0 and *E^ij^*(1, 0) − *E^ij^*(1, 1) > 0. Similar constructions for other case exists and we refer the reader to Kolmogorov and Zabin (2004) for more details. The complete graph is built by merging the elementary graphs for each node pair as illustrated in Fig. 3(b). After the complete graph is built the minimum cut on it can be found by pushing the maximum flow between the source and sink.

Phase measurements in low signal areas tend to be less reliable and these areas can be discounted by assigning a weight to each voxel based on its magnitude. Similar to Ying et al. (2006), we use the magnitude image as a quality map and assign greater weight to voxels having large magnitude values.

After the phase images are unwrapped, the deformation field to correct the EPI image can be computed through Eqs. (3) and (4). However, as previously mentioned, the estimated deformation can be inaccurate in image areas with low signal. In the following section, we describe a way to compute the uncertainty associated with the estimated fieldmap and how this uncertainty information can be used in conjunction with an image registration step to further improve the results.

## 5 Uncertainty estimation and image registration

This section explains how we can combine the fieldmap correction technique described in the previous step with image registration based techniques. The two techniques can be unified by estimating the uncertainty from the fieldmap step and using it with image registration to refine the deformation in image areas where the estimated fieldmap is likely to be inaccurate. The following sub-section describes how uncertainty information can be estimated during the phase unwrapping step.

### 5.1 Uncertainty estimation in phase unwrapping

Besides fast MAP inference, another advantage of using graph cuts is its ability to be able to generate the uncertainty associated with the most likely MRF configuration. It was shown by Kohli and Torr (2008) that the uncertainty associated with the MAP solution can be estimated using graph cuts through computation of *max-marginals*. Max-marginals are a general notion and can be defined for any function as Eq. (12). Hence, the max-marginal (*α*_*v*;*j*_) is the maximum probability over all possible MRF configurations where an MRF site *x_v_* is constrained to take the label *j* (*x_v_* = *j*).

(12) $$\alpha_{v;j}=\underset{x\in L,x_{v}=j}{\text{max}}P\left(x|Y\right)$$

The max-marginals can be used to compute the confidence measure (*ω*) associated with any random variable labelling as Eq. (13).

(13) $$\omega_{v;j}=\frac{{\text{max}}_{x\in L,x_{v}=j}P\left(x|Y\right)}{\sum_{k\in L}{\text{max}}_{x\in L,x_{v}=k}P\left(x|Y\right)}=\frac{\alpha_{v;j}}{\sum_{k\in L}\alpha_{v;k}}$$

Therefore, the confidence *ω*_*v*;*j*_ for a random variable *x_v_* to take the label *j* is given by the ratio of the max-marginal associated with assigning label *j* to variable *x_v_* to the sum of max-marginals for all possible label assignments for the variable *x_v_*.

As shown by Kohli and Torr (2008), this confidence can be expressed in terms of *min-marginal* energies. Min-marginal (*ψ*) is the minimum energy obtained when we constrain a random variable to take a certain label and minimise over all the remaining variables as in Eq. (14).

(14) $$\psi_{v;j}=\underset{x\in L,x_{v}=j}{\text{argmin}}\;E\left(x\right)$$

The energy and probability of a labelling configuration are related through the expression for Gibbs energy function as:

(15) E(x)=−logP(x|Y)−logZ

where *Z* is the partition function. Substituting the value of *P*(*x*|*Y*) in Eq. (12) we have:

$$\begin{array}{l} \alpha_{v;j}=\underset{x\in L,x_{v}=j}{\text{max}}\left(\text{exp}\left(-E\left(x\right)-\text{log}\;Z\right)\right)\\ \;=\frac{1}{Z}\text{exp}\;(\underset{x\in L,x_{v}=j}{-\text{argmin}}E\left(x\right)) \end{array}$$

Finally substituting Eq. (14), we have:

(17) $$\alpha_{v;j}=\frac{1}{Z}\text{exp}\left(-\psi_{v;j}\right)$$

Note that the knowledge of the partition function is not necessary to compute the max-marginal confidence measure. As an example, let us consider computing the max-marginal for a voxel to take a certain label 0. For the sake of simplicity, let us assume that it is a binary problem and only two configurations for this voxel are possible namely 0 and 1. The max-marginal value for this voxel to take the label 0 is given by:

(18) $$\omega_{v;0}=\frac{\frac{1}{Z}\text{exp}\left(-\psi_{v;0}\right)}{\frac{1}{Z}\text{exp}\left(-\psi_{v;0}\right)+\frac{1}{Z}\text{exp}\left(-\psi_{v;0}\right)}$$

Note that the *Z*’s cancel out from the numerator and denominator.

Hence, the confidence measure (*ω*_*v*;*j*_) associated with any random variable *x_v_* to take the label *j* can be expressed in general terms as Eq. (19), without estimating the partition function *Z*.

(19) $$\omega_{v;j}=\frac{\text{exp}\left(-\psi_{v;j}\right)}{\sum_{l\in L}\text{exp}\left(-\psi_{v;l}\right)}$$

Dynamic Graph Cuts can be used to compute *ω*_*v*;*j*_ for each voxel at every binary optimisation step in a very efficient manner. A given MRF node can be constrained to belong to the source or the sink by adding an infinite capacity edge between it and the respective terminal node. No other changes need to be made to the graph and the required min-marginal can be computed by optimising the resulting MRF. Hence, to compute the min-marginals at every binary optimisation step, one has to optimise one such MRF for every node *v* and each of the two labels. Usually these MRFs are very close to each other and form a slowly varying dynamic MRF system, which means that the search trees from previous computations can be efficiently reused, which greatly reduces the computation time.

This confidence map generated from the phase unwrapping step gives us a way to combine field map and image registration based susceptibility artefact correction techniques in an intuitive way. Areas of high uncertainty from the phase unwrapping step indicate where the generated field map is more likely to be unreliable. This knowledge can be used to refine the results in these areas using image registration. The following section describes how the generated deformation field and the confidence map can be used in an image registration framework to further improve the results.

### 5.2 Image registration framework

The displacement field and the confidence map generated from the phase unwrapping step are used to initialise the subsequent non-rigid registration step. As discussed in the introduction, registration between the distorted EPI images and the undistorted T1/T2 weighted MR images is a popular alternative to using field maps for correcting for susceptibility artefacts. In this section we will show how the two approaches can be combined using the uncertainty information derived from the phase unwrapping step.

The registration algorithm we developed follows closely from Glocker et al. (2008), So et al. (2011) and is formulated as a discrete multi-labelling problem. The deformation field is parameterised using cubic B-splines as in Rueckert et al. (1999), Modat et al. (2010) which has the desirable property of generating deformations that are *C*^2^ continuous. The basic idea is that a uniformly spaced cubic spline control point mesh is overlaid on the image. A spline control point controls the position of certain voxels in its neighbourhood. So, by perturbing the control points, local deformations can be induced in the image.

A mutual information based image similarity measure was chosen for the proposed image registration algorithm. The key advantage of mutual information based measures is their ability to easily handle complex relationships between the intensities in the two images. They require no a-priori model of the relationship between the image intensities and can handle image registration between different modalities. Typically, graph cuts based optimisation algorithms cannot use such global similarity measures in the optimisation as it is difficult to adapt them directly in the data term in Eq. (8). To overcome this problem, a local variant of normalised mutual information (SEMI) as described by Zhuang et al. (2011) is used as the similarity measure. SEMI computes mutual information in a local region with respect to each of the control points. However, it uses a hierarchical weighting scheme to differentiate the contributions of different voxels to the similarity measure. The weighting scheme is chosen such that the weight given to a voxel is monotonically decreasing with respect to the distance between the voxel and the spline control point. Under this scheme, the joint histogram is computed as shown in Eq. (20) where *I_r_*(*x*) and *I_f_*(*x*) are the reference and transformed floating images. *w_r_* and *w_f_* are Parzen windows functions and the joint histogram is calculated for the local region *Ω_s_*. *Γ_s_*(*x*) is a weighting function for the spatial encoding and is a Gaussian kernel centred on the control point. Hence, local joint histograms are computed for each of the control points and the corresponding data term used is generated by computing the normalised mutual information (Studholme et al., 1999) from each of these joint histograms. The local nature of the similarity measure allows the problem to be formulated in the MRF framework which can be solved using the graph cuts framework.

(20) $$H_{s}\left(r,f\right)=\sum_{x\in \Omega_{s}}\left(w_{r}I_{r}\left(x\right)w_{f}I_{f}\left(x\right)\right)\Gamma_{s}\left(x\right)$$

As registration is an ill-posed problem, priors on the estimated deformation field is usually introduced in the form of a smoothness term. A simple smoothness term would be to use the magnitude of the displacement vector difference at every registration iteration. This would result in registration scheme where incremental updates to the deformation field are penalised. This update scheme has the advantage of fulfilling the inequality constraint of Eq. (9) and can be easily accommodated into the graph cuts framework. However, it does not provide a regularisation over the full time course of the registration. In this work, we penalise the magnitude of the difference in the deformation as in Glocker et al. (2008) to perform a full regularisation as:

(21) $$E_{ij}\left(\chi_{i},\chi_{j}\right)=\left|\left(ℛ\left(i\right)+d_{i}\right)-\left(ℛ\left(j\right)+d_{j}\right)\right|$$

where ℛ(.) projects the current displacement field to the control points and *d* is the displacement updates for the current iteration. It is worth noting that the inequality constraint of Eq. (9) for the pairwise term is not guaranteed to be met anymore. However, this is rarely a problem in practice as demonstrated in Glocker et al. (2008). The MRF nodes where the edge weights turn negative and the inequality constraint was violated were handled by setting those pairwise edge weights to zero. In practice, this condition was only encountered in a handful of voxels.

The geometric distortion due to susceptibility is dominant in the phase encode (PE) direction. Hence the B-spline control points are constrained to move only in the PE direction. A discrete set of displacements is considered in the PE direction and a label assignment to a control point is associated with displacing the control point by the corresponding displacement vector. Therefore, registration is done by solving this discrete multilabel problem modelled in the first-order MRF, where the cubic B-spline control points are the random variables and the goal is to assign individual displacement values to these nodes.

The final task that remains is to integrate the uncertainty information from the field map estimation step into the registration framework. The registration is initialised with the deformation field obtained from the field map. The goal is to drive the registration in areas where the field map results are uncertain. This is achieved by modulating the weight of the global penalty term (*λ* in Eq. 22) by the confidence map obtained during the phase unwrapping step. This local modulation factor is computed at each control point as a weighted sum of the confidence values from the voxels that are influenced by the control point, where the weighting kernel is computed from the current B-spline deformation grid. This has the affect of keeping the weight of the penalty term high in regions where the fieldmap is estimated with a low level of uncertainty thus discouraging large displacements whilst relaxing it in regions of high uncertainty to allow for more displacement. Hence, the spatially varying cost function takes the form of Eq. (22) where *σ_i_* is the spatially varying confidence at voxel *i*, *λ* is the global penalty term weight and SEMI_*i*_ is the unary data term at control point *i*. The pairwise term *E_ij_*(*x_i_*, *x_j_*) is as defined in Eq. (21). The penalty term weights are initialised by projecting the confidence map on the control point grid. This cost function is optimised using an *α*-expansion variant of the graph cuts minimisation algorithm (Boykov et al., 2001).

(22) $$E=-\sum_{i\in I}\left[(1-\sigma_{i}\lambda )\times {\text{SEMI}}_{i}]+[\sigma_{i}\lambda \times E_{ij}(x_{i},x_{j})\right]$$

Similar to Studholme et al. (2000), the intensity distortions, due to susceptibility artefacts, are taken into account by recomputing the EPI intensities during image registration as *I_f_* = *I_Tf_J_T_* where *I_f_* is the Jacobian corrected EPI image in the space of the reference anatomical image, *I_Tf_* is the transformed EPI image where *T* is the current estimate of the transformation and *J_T_* is its Jacobian determinant.

## 6 Validation

### 6.1 Validation using simulated data

We validated the phase unwrapping algorithm using simulated phase MRI data. To conduct the simulations, an MRI simulator software package was used: POSSUM (Physics-Oriented Simulated Scanner for Understanding MRI) (Drobnjak et al., 2006; Drobnjak et al., 2010). POSSUM is a simulator which generates realistic MR images. The simulator achieves this by simulating an MR scanner with various scanner input parameters operating on a physical model of the brain. The output of the simulator is the signal received from the receiver coil of the simulator scanner. The algorithm solves fundamental Bloch equations to model the behaviour of the magnetisation vector for each voxel of the brain and for each tissue type independently. The signal coming from one voxel is obtained by analytical integration of magnetisation over its spatial extent, and the total signal is formed by numerically summing the contributions from all the voxels. For a given brain phantom, pulse sequence and magnetic field values, POSSUM generates realistic MR images. Magnetic field values are calculated by solving Maxwell’s equations which as an input use an air-tissue segmentation of the brain, and their respective susceptibility values. These magnetic field values are fed into the Bloch equation solver in POSSUM, resulting in images with realistic susceptibility artefacts. A further, in-depth description of POSSUM is presented in Drobnjak et al. (2006).

Here we use a 3D digital brain phantom from the MNI Brain-Web database, which is thoroughly segmented into various tissues such as grey and white matter, cerebrospinal fluid, and has a good air-tissue segmentation (Collins et al., 1998). We assume a 1.5 T scanner, and use appropriate MR parameter values for white matter (*T*_1_ = 833 ms, *T*_2_ = 83 ms, spin density *ρ* = 0.86); (grey matter *T*_1_ = 500 ms, *T*_2_ = 70 ms, *ρ* = 0.77) and CSF (*T*_1_ = 2569 ms, *T*_2_ = 329 ms, *ρ* = 1). A typical fieldmap sequence was simulated: two gradient echo images with different echo times (*TE*_1_ = 8 ms; *TE*_2_ = 10 ms). Spatial resolution was 2.5 mm × 2.5 mm × 2.7 mm and *TR* = 700 ms.

**Fig. 4:**
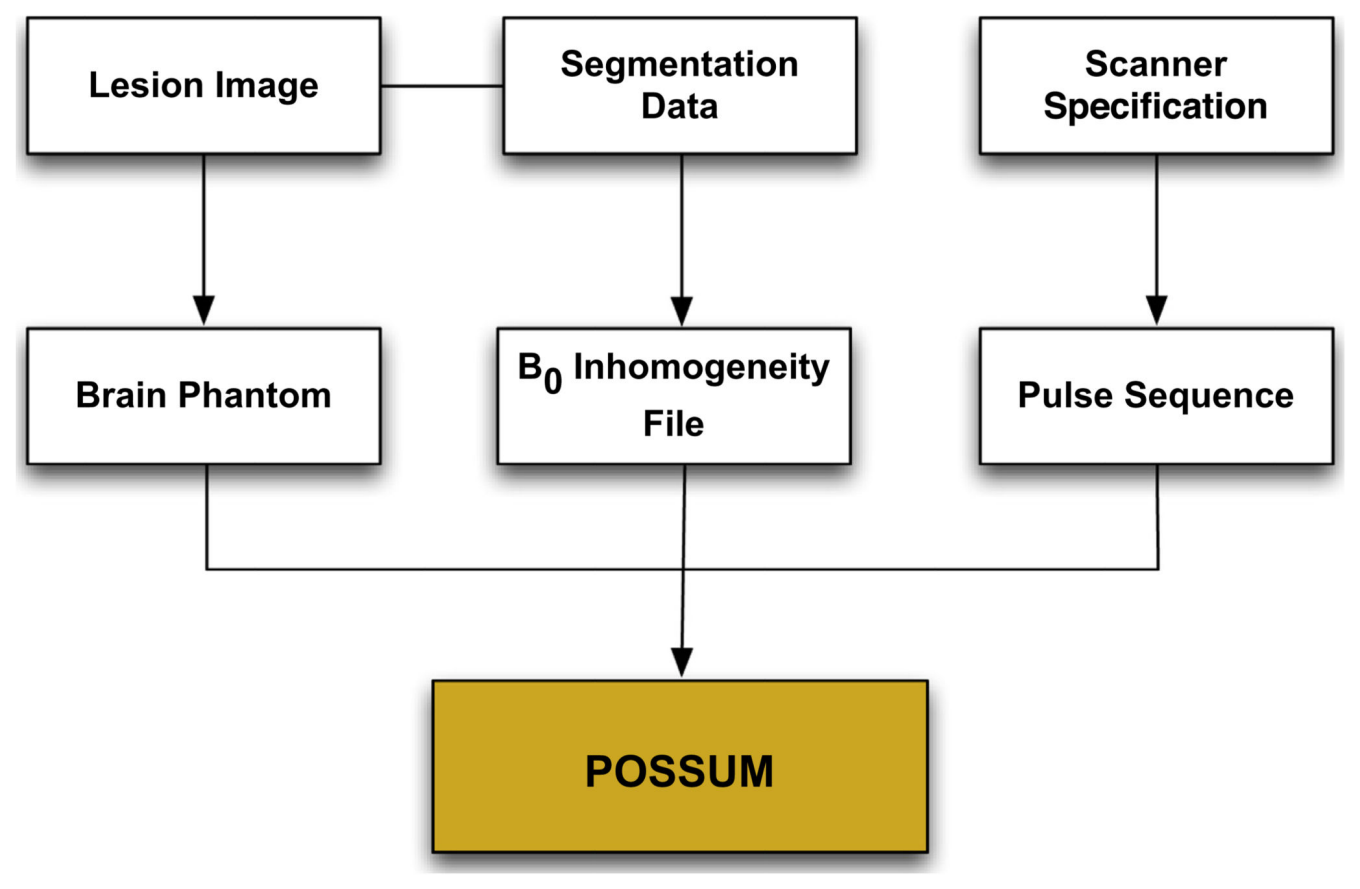
The various inputs to POSSUM to simulate the MRI phase images. Lesions are manually drawn in the input phantom image. The *B*_0_ inhomogeneity file describes change in magnetic field strength inside the cranium due to tissue susceptibility differences. To calculate these distortions, Maxwell’s equations are solved at each voxel in an air-tissue segmentation volume using the perturbation method. Finally, the MRI pulse sequence (eg. EPI) characteristics can be specified for each simulation.

**Fig. 5:**
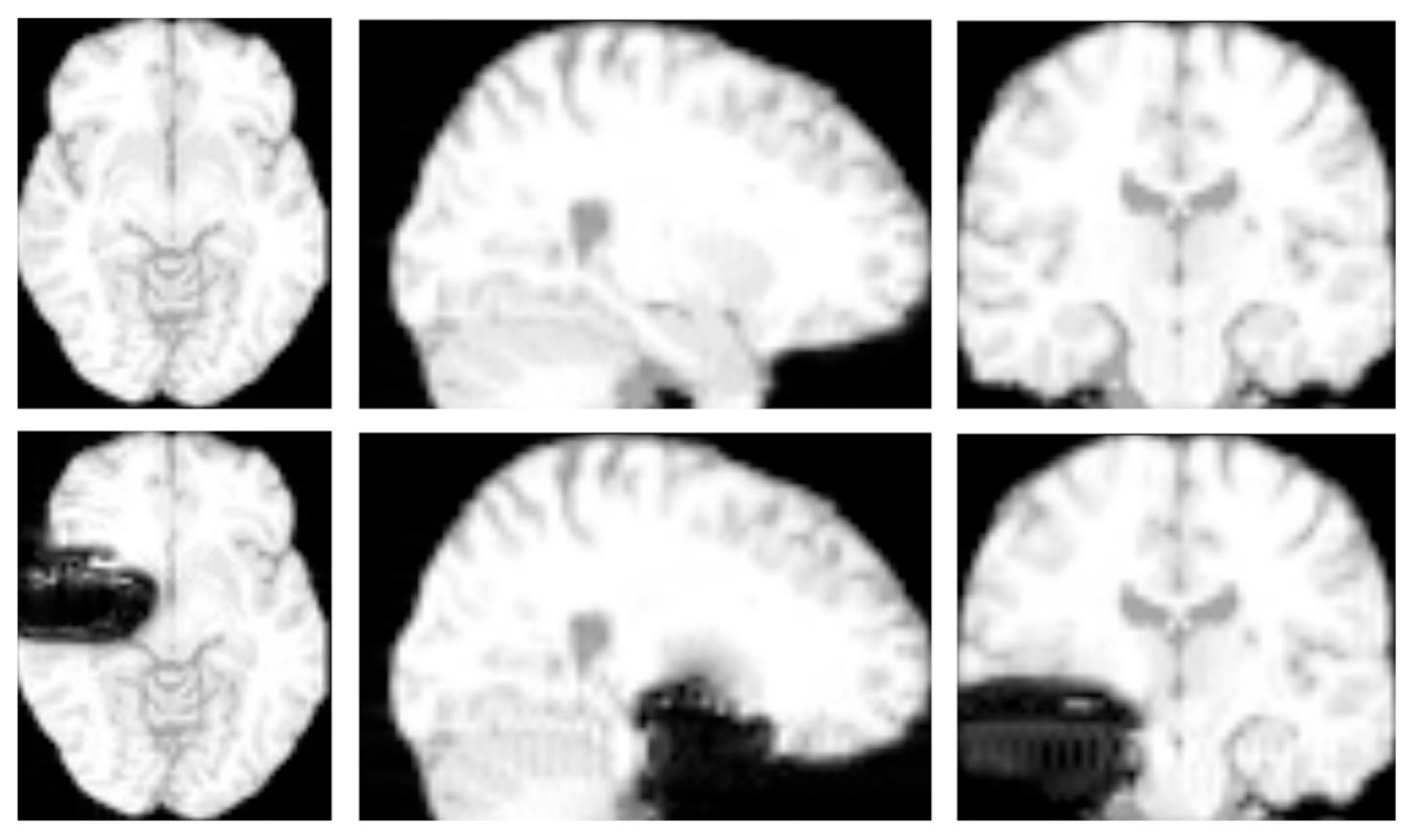
Example images produced by the POSSUM simulator. The top row shows a simulated gradient echo MRI image. The bottom row shows the image with the simulated surgical resection.

In order to make the simulated images representative of images acquired during a surgical procedure, resections were introduced into the input phantom. The resections were designed to match the typical resections made during anterior temporal lobe resection for refractory epilepsy. Hence, actual T1-weighted intra-operative scans were used as reference for resection design. This modified phantom was used as an input to POSSUM and wrapped phase images and ground truth magnetic field values were simulated. The various inputs to the POSSUM simulator is shown in Fig. 4. Example images generated by the simulator are shown in Fig. 5.

For the validation, various levels of Gaussian noise were added to the ground truth unwrapped phase images. The corrupted images were then wrapped back to generate the phase images to be used as input for the unwrapping algorithms. For comparison, the images were unwrapped using the proposed unwrapping algorithm as well as with PRELUDE (Jenkinson, 2003), a freely available software package available with FSL (Smith et al., 2004) and widely used within the neuroimaging community.

**Fig. 6:**
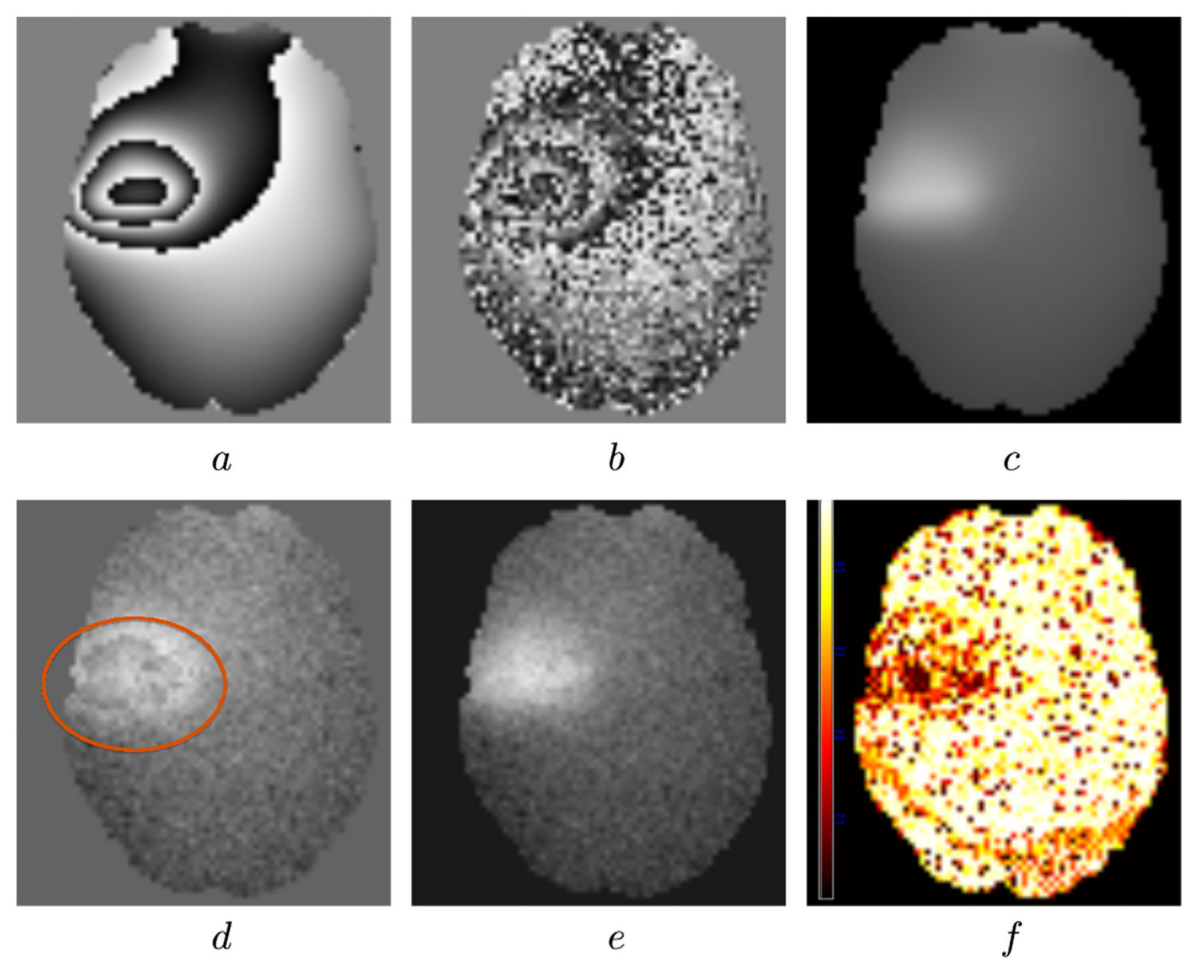
Results from phase unwrapping. (a) Is a masked slice through a noise free wrapped image. (b) Is the same image where the ground truth unwrapped image was corrupted with Gaussian noise. (c) Shows the ground truth unwrapped image. (d) Shows the unwrapping result from PRELUDE. Some areas with phase discontinuities are visible in the unwrapped result (highlighted in red). (e) Shows the unwrapped image using the proposed phase unwrapping algorithm where no phase discontinuities are evident. (f) Shows the confidence map obtained using the proposed algorithm. Darker regions indicate low confidence areas where we are less certain about the quality of our unwrapping. (For interpretation of the references to colour in this figure legend, the reader is referred to the web version of this article.)

The quantitative unwrapping results for the proposed method and PRELUDE are shown in Table 1. The results were compared with the original (ground truth) phase, and the misclassification ratio (MCR) was calculated. The MCR is the ratio of the number of voxels that were incorrectly unwrapped to the total number of voxels. Both PRELUDE and the proposed unwrapping algorithm perform comparably well under low-noise conditions. However, at higher noise levels the proposed algorithm outperforms PRELUDE both in terms of MCR and execution time. In addition, the proposed algorithm also generates the confidence associated with the unwrapping solution and can compute it within the time constraints associated with a neurosurgical procedure. A visual example is shown in Fig. 6. Some discontinuities around the lesion still exist when unwrapping with PRELUDE but not when using the proposed phase unwrapping technique. Fig. 6(f) shows the confidence map generated along with the unwrapped image.

### 6.2 Validation using clinical data

We used the proposed algorithm on 13 datasets that were acquired using interventional MRI during temporal lobe resection procedures for surgical management of temporal lobe epilepsy. The imaging was done as part of an audit to quantify the benefits of using iMRI on patient outcome for subjects having temporal lobe resections. The images were acquired using a 1.5 T Espree MRI scanner (Siemens, Erlangen) designed for interventional procedures. The T1 MR image, used in the registration step, had a resolution of 1.1 × 1.1 × 1.3 mm using a 3D FLASH sequence with *TR* = 5.25 ms and *TE* = 2.5 ms. The EPI images used a single shot scheme with GRAPPA parallel imaging (acceleration factor of 2) and had a spatial resolution of 2.5 × 2.5 × 2.7 mm. The noise variance in these datasets was measured in manually selected region of interest known to only contain air. The mean noise variance was 0.71 radians.

Validation of the proposed susceptibility correction in the absence of ground truth deformation is not trivial. A popular approach has been to identify landmarks on the EPI and T1 or T2 MR images (obtained with conventional spin or gradient echo sequences with negligible spatial distortion) and measure the distance between the landmarks before and after performing the correction. However, this method tends to bias the results towards image registration based schemes. This is because intensity based registration algorithms tend to perform better in regions with high contrast which is precisely where landmarks can be reliably identified. Secondly, it is very difficult to reliably pick landmarks on interventionally acquired EPI images due to increased levels of noise, low spatial resolution and presence of deformation. Since we are interested in achieving accurate artefact correction in the white matter areas, we focused on looking at the effect of susceptibility correction on residual tensor fit errors. One significant source of tensor fit errors is the geometric distortions arising from susceptibility artefacts. Hence, accurate correction of susceptibility artefacts should reduce residual errors after performing tensor fitting. A previous study also demonstrated that nonlinear correction of susceptibility artefacts resulted in smaller tensor fit errors (Kim et al., 2006).

The normalised sums of square of diffusion tensor fit errors (*χ*^2^) is given by Eq. (23) where *N* signals are fitted and *S_m_* and *S_f_* are the measured and fitted signals respectively (Papadakis et al., 2002).

(23) $$\chi^{2}=\frac{\sum_{i=1}^{N}{\left(S_{m}-S_{f}\right)}^{2}}{\sum_{i=1}^{N}S_{m}^{2}}$$

**Fig. 7:**
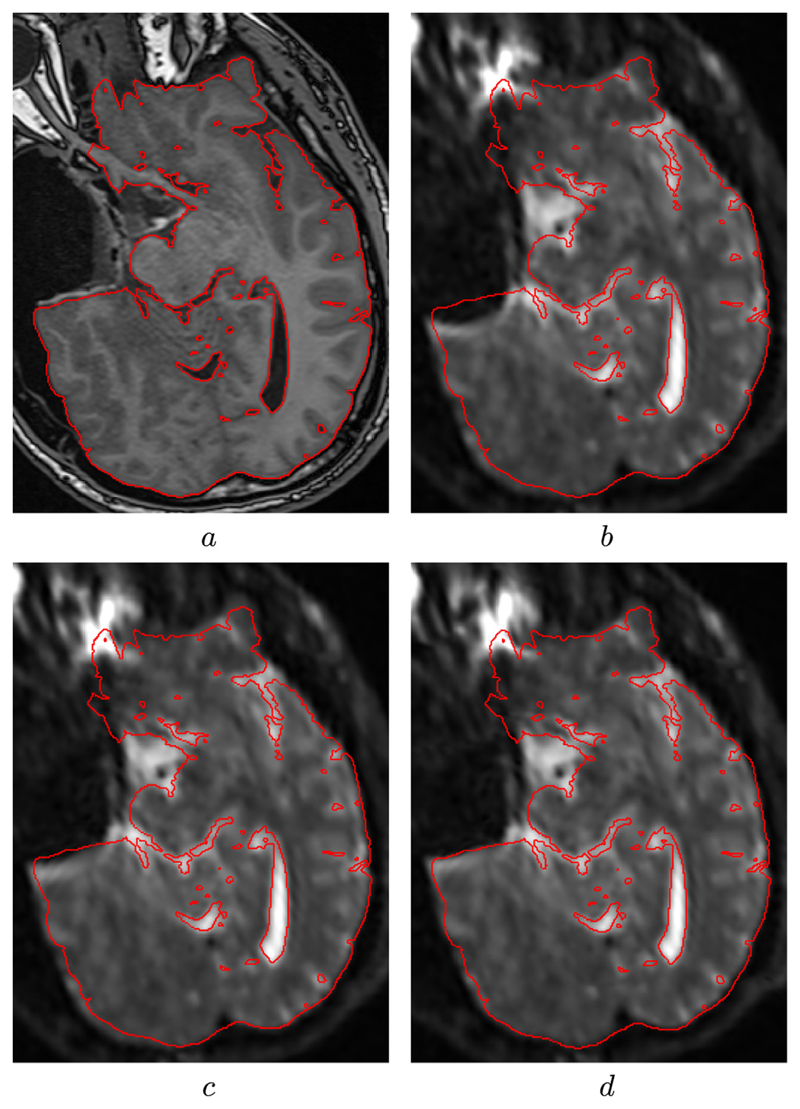
Images showing the result of correcting for susceptibility-induced spatial distortion using our algorithm. (a) Shows the gold-standard high resolution T1 image acquired during surgery. (b) Shows the uncorrected B0 image with a large geometric distortion around the resected area. (c) Shows the result of correcting for susceptibility artefacts using the proposed fieldmap estimation. (d) Shows further improvement in the result when combined with the image registration step.

The diffusion tensors were reconstructed using *dtifit* (Smith et al., 2004) and sum of square residual errors for the diffusion tensor fits were obtained for the 13 subjects. For the validation, the initial sums of square residual tensor fit errors were computed for all subjects. Correction was performed after unwrapping the phase maps using PRELUDE and the proposed phase unwrapping algorithm. We also performed the correction using the registration algorithm described in Section 5.2 and finally using the proposed method combining the fieldmap and image registration algorithm. The quantitative results are described in Table 2. A paired t-test showed that the proposed method showed a statistically significant reduction (p-value < 10^−3^) in residual tensor fit errors when compared to fieldmap and image registration based techniques alone. Fig. 7 shows a representative slice where the corrected B0 image using the proposed method shows good visual correspondence with the undistorted T1-weighted image. The mean computation time for the proposed method for these 13 subjects was 2 min 32 s, making it suitable to be used during neurosurgery.

Based on these results, the proposed method has been integrated into the surgical workflow at NHNN and is used as a pre-processing step before performing the non-rigid registration proposed in Daga et al. (2012) to localise the Optic Radiation during surgery.

All validation was performed on a standard workstation with 16 2 GHz CPU cores and 32 GB of physical memory. The software was written using standard C++ and can be run under multiple operating systems.

## 7 Discussion and conclusion

Accurate susceptibility correction is important to make effective use of diffusion imaging capabilities of interventional MRI. Susceptibility artefacts are especially severe in the interventional setting due to the presence of resection cavity which induces large susceptibility differences. The problem is further amplified by the fact that the wide-bore MR systems often used in intra-operative applications have a smaller uniform static magnetic field region than diagnostic scanners and the subject head usually does not experience a uniform magnetic field as there is limited flexibility with regards to the head placement. This problem can be mitigated to a certain degree by filling the resection cavity with a saline solution, but this is not possible in a majority of operations due to inconvenient head placement, which allows the saline solution to fall out of the resection cavity. The extent of distortion can also be reduced by use of parallel imaging techniques (Pruessmann, 2006) or segmented EPI acquisition (Atkinson et al., 2000). However, this is not always feasible and does not completely eliminate the distortions.

In this study, we have considered the confidence associated with the phase unwrapping solution and used this information to in a subsequent image registration step to further improve the results in areas of low confidence. The results show that this hybrid approach leads to better fit of the tensor model to the DWI images suggesting that the correction of susceptibility artefacts can improve the usability of such images in the neurosurgical setting. For future work, we also aim to develop regularised fieldmap estimation techniques that can account for noise in phase maps in regions of low spin density and also utilise the fact that fieldmaps tend to be piecewise smooth. Conventionally, the fieldmap is smoothed using a low pass filter but this can further propagate the errors especially when the measured phase is severely corrupted. Another useful investigation would be to combine susceptibility correction with correction of other MRI artefacts arising from eddy currents and vibration. A limitation of this work is that the estimated min-marginals to characterise confidence in the phase unwrapping solution are not exact marginal probabilities. A useful addition would be to use inference schemes like Markov Chain Monte Carlo or approximate ones like Variational Bayes or Expectation Propagation to characterise the posterior distributions and generate exact marginal probabilities. However, currently this cannot be done within the time constraints of a neurosurgical procedure.

Accurate measurement of phase is critical in various other contexts in MRI like flow imaging and susceptibility weighted imaging (SWI). SWI exploits the magnetic susceptibility differences between various tissues and the phase images generated from SWI are useful for detection of cerebral microbleeds in patients with traumatic brain injuries (Haacke et al., 2009). SWI requires long echo times and suffers from severe phase wraps especially in regions of sharp tissue susceptibility differences. This proposed phase unwrapping algorithm is fast enough to be implemented directly in the scanner acquisition and image creation pipeline.

In summary, we have combined fieldmap and image registration based correction approaches by estimating the confidence associated with the phase unwrapping step and incorporating it in the subsequent registration step. We have shown that the proposed unified technique performs better than using fieldmaps or image registration based techniques alone when used on interventionally acquired EPI images on a dataset of thirteen subjects. This work has been integrated in the surgical workflow at NHNN and is being used to facilitate effective neurosurgical treatments.

## Figures and Tables

**Table 1:** Misclassification ratio (MCR) and execution time (in seconds) for generating the fieldmap from the synthetic phase images. The MCR is defined as the ratio between the voxels that were incorrectly wrapped to the total number of voxels. For small amounts of phase noise (noted in radians), both the proposed phase unwrapping algorithm and PRELUDE perform similarly. However, for larger noise levels, the proposed algorithm results in lower MCR. The execution time of PRELUDE for high levels of phase noise does not satisfy the stringent time requirements of neurosurgery, while the proposed algorithm executes well within the time constraints. Time-1 refers to the time taken by the proposed method to do phase unwrapping without confidence map estimation. Time-2 is for phase unwrapping along with confidence map estimation. All times are reported in seconds. The mean noise variance in the standard clinical datasets produced on the iMRI was 0.71 radians (corresponding simulation result highlighted in bold).

Noise variance (rad.)	0.08	0.26	0.52	**0.71**	0.87	1.0	1.2
MCR (proposed)	0.01	0.04	0.12	**0.12**	0.15	0.19	0.26
MCR (PRELUDE)	0.01	0.06	0.17	**0.19**	0.21	0.27	0.31
Time-1 (proposed) sec.	4	4	6	**9**	9	8	9
Time-2 (proposed) sec.	23	22	24	**24**	28	30	32
Time (PRELUDE) sec.	4	17	154	**472**	1520	2213	4276

**Table 2:** Mean(standard deviation) of the sum of square errors for diffusion tensor fitting in interventionally acquired diffusion weighted images for thirteen patients. The first column (Initial) shows the initial mean error. The second column (PRELUDE) shows the fit errors after correcting for susceptibility artefacts using PRELUDE. The third column (Fieldmap only) shows the tensor fit errors after correcting for susceptibility artefacts using the fieldmap generated after unwrapping the phase maps using the proposed phase unwrapping algorithm. The fourth columns (Reg. only) shows the tensor fit errors after correcting for susceptibility artefacts using the proposed registration algorithm. The final column (Proposed) shows the tensor fit errors after combining the fieldmap and image registration methods using the proposed method. The proposed method showed statistically significant improvement over the other methods (*p*-value < 10^-3^). The final row shows the mean tensor fit errors and standard deviation over all the cases.

Initial	PRELUDE	Fieldmap only	Reg. only	Proposed
3.08(1.94)	1.92(1.14)	1.51(1.23)	1.31(0.97)	1.23(0.84)
2.94(1.89)	1.51(1.42)	1.48(1.35)	1.14(0.87)	1.12(0.73)
2.97(3.56)	1.94(2.26)	1.94(2.26)	1.98(1.56)	1.03(1.21)
3.40(3.17)	2.71(1.54)	2.42(1.06)	2.51(1.99)	2.34(0.98)
1.76(1.42)	1.43(1.36)	1.38(1.11)	1.12(0.76)	1.08(0.81)
2.27(2.30)	1.23(1.08)	1.36(1.02)	1.68(1.54)	1.22(1.12)
3.85(3.91)	2.78(2.51)	2.42(2.02)	2.53(1.91)	2.39(1.62)
2.70(2.37)	2.12(1.43)	2.04(1.51)	2.04(1.63)	1.72(1.43)
3.60(3.51)	2.53(2.01)	2.19(1.84)	2.61(2.30)	1.81(0.93)
2.32(1.85)	1.32(1.01)	1.45(1.04)	1.76(1.34)	1.41(0.96)
2.17(2.11)	1.16(0.86)	1.12(0.72)	1.47(1.14)	1.07(0.93)
2.81(2.62)	1.93(1.62)	1.59(1.22)	2.12(1.69)	1.41(1.04)
2.02(2.17)	1.16(0.91)	1.07(0.86)	1.41(1.38)	1.01(0.92)
				
2.76(0.63)	1.82(0.58)	1.69(0.46)	1.82(0.52)	1.44(0.47)
